# Psychiatric outcomes in the first two years after the October 7 attacks: a systematic review of posttraumatic stress, anxiety, depression, and addiction-related outcomes

**DOI:** 10.3389/fpsyt.2026.1883498

**Published:** 2026-09-09

**Authors:** Michal Cohen, Maor Daniel Levitin, Or Gliksberg, Einav Levine, Tslil Dvir, Mario Mikulincer

**Affiliations:** 1 Israel Center for Addiction and Mental Health (ICAMH), Department of Psychology, The Hebrew University of Jerusalem, Jerusalem, Israel; 2 School of Psychological Sciences, Tel Aviv University, Tel Aviv, Israel

**Keywords:** addiction, anxiety, collective trauma, depression, posttraumatic stress symptoms (PTSS), systematic review

## Abstract

**Objectives:**

The October 7 attacks and ensuing war created conditions of sustained, multidimensional trauma. This systematic review synthesizes studies from the first two years of the war to clarify early prevalence of post-traumatic stress symptoms (PTSS), anxiety, depression, and addiction-related outcomes in the Israeli population.

**Methods:**

Following PRISMA 2020 guidelines, PubMed, PsycINFO, Scopus, and Web of Science were searched identifying studies published between October 7, 2023 and October 7, 2025 (final search date December 30, 2025). Guided by the PICOS framework, eligible studies assessed psychiatric outcomes among Israeli populations exposed to the October 7 attacks or subsequent war. Outcomes of interest included posttraumatic stress, anxiety, depression, and addiction-related outcomes, and observational quantitative and qualitative studies were included. Risk of bias was assessed using Joanna Briggs Institute tools. Findings were synthesized narratively, with attention to exposure characteristics, subgroup differences, and time of assessment.

**Results:**

Sixty-eight studies met inclusion criteria. Symptom trajectories were heterogeneous and varied with exposure intensity and contextual vulnerability. Elevated PTSS was associated with direct, cumulative, and role-related exposure. Anxiety and depression were widely documented and persisted among individuals facing sustained threat, socioeconomic strain, or displacement. Addiction-related outcomes, an emerging and comparatively understudied domain, were examined in only three studies, with preliminary evidence suggesting exposure and distress-associated increases in substance use and gambling.

**Conclusions:**

Psychiatric responses following October 7 were domain-specific and associated with exposure, ongoing stress, and structural and individual vulnerabilities, underscoring the need for targeted, longitudinal monitoring in prolonged crisis contexts.

## Introduction

The Hamas-led attacks of 7 October 2023 and the subsequent war between Israel and Hamas (“Iron Swords”) constitute a large-scale collective trauma with substantial implications for population mental health (e.g., 1). Compared to prior escalations in the region, the magnitude of civilian casualties, geographic breadth of exposure, mass displacement, and prolonged uncertainty created sustained, multidimensional threat conditions affecting large segments of the Israeli population (1). These circumstances raise urgent questions regarding the nature, distribution, and clinical significance of emerging psychiatric responses following prolonged collective violence. Beyond the immediate regional context, understanding psychiatric responses under conditions of sustained societal threat has broader relevance for mental-health research and policy in settings of protracted conflict and large-scale crises worldwide. In the months following October 7, empirical research expanded rapidly. Cross-sectional surveys, longitudinal cohorts (including pre-war baselines), and studies of civilian, military, and other high-risk subgroups began documenting psychiatric outcomes under conditions of ongoing threat (e.g., 2–7). This proliferation provides a rare opportunity to examine early psychiatric responses in near real time. However, the literature is characterized by substantial heterogeneity in exposure definitions, sampling strategies, assessment timing, and measurement instruments, challenges commonly observed in disaster mental health research (8). As a result, findings remain fragmented across symptom domains and population groups, limiting integrative understanding and complicating evidence-based clinical and policy decision-making.

A differentiated synthesis of distinct symptom domains is necessary to distinguish transient stress reactions from patterns that may signal risk for persistent psychopathology (9). The present review therefore focuses on four domains consistently assessed in the emerging literature: posttraumatic stress symptoms (PTSS), anxiety, depression, and addiction-related outcomes, including problematic substance use and behavioral addictions. PTSS represent trauma-linked symptoms directly associated with exposure to life-threatening events (10). Anxiety and depressive symptoms, although not trauma-specific, are robust predictors of functional impairment and long-term morbidity following large-scale crises (11). Addictive behaviors have been theoretically and empirically associated with trauma exposure (e.g., 12) and affective dysregulation (13, 14) and may emerge as maladaptive coping responses under sustained threat (15, 16). However, addiction-related outcomes remain an emerging and comparatively understudied domain in research on large-scale collective trauma.

In addition to variability in symptom domains, exposure to the October 7 attacks and subsequent war has been highly heterogeneous, encompassing direct life-threatening experiences, diverse forms of indirect exposure (e.g., bereavement, displacement, repeated missile alerts, and prolonged media exposure), cumulative stressors, and role-related exposure (e.g., civilians, reservists, evacuees). Accordingly, this review provides the first systematic synthesis of psychiatric outcomes across multiple symptom domains during the first two years following the October 7 attacks. It remains unclear whether the elevated symptom levels reported across studies reflect a uniform rise affecting all domains to a similar degree, or domain-specific elevations in which particular exposure pathways are associated with particular outcomes.

The present systematic review synthesizes empirical studies published during the first two years after the October 7, 2023, attacks. By integrating evidence on prevalence, exposure characteristics, and subgroup differences across PTSS, anxiety, depression, and addictions, this review aims to characterize early prevalence patterns and to delineate how distinct forms of exposure relate to domain-specific psychiatric responses. In doing so, it seeks to inform targeted clinical screening, resource allocation, and public mental health planning under conditions of prolonged collective crisis.

## Methods

### Protocol and reporting standard

This systematic review was conducted in accordance with the Preferred Reporting Items for Systematic Reviews and Meta-Analyses (PRISMA) 2020 statement (17). Reporting was guided by the PRISMA 2020 checklist and flow diagram. The review was not prospectively registered. A written review protocol specifying the objectives, eligibility criteria, search strategy, and synthesis plan was prepared before screening commenced, reducing the risk of *post-hoc* selective reporting. The review was not, however, prospectively registered in a public registry such as PROSPERO. This was a full systematic review, not a rapid review. Although it was initiated promptly in response to an evolving situation, all steps followed standard systematic-review procedures. The absence of prospective registration is acknowledged as a limitation, although the pre-specified protocol, finalized before screening began, mitigates the associated risk of selective reporting.

### Review aim

The aim of this review was to synthesize empirical evidence on posttraumatic stress symptoms (PTSS), anxiety, depression, and addictions, including problematic substance use and behavioral addictions, among Israeli populations during the first two years of the Iron Swords since October 7, 2023. Particular attention was given to exposure characteristics, timing of assessment, and differences across demographic and role-defined subgroups in order to clarify domain-specific patterns of psychiatric responses.

### Eligibility criteria

Studies were eligible for inclusion if they:

1. Employed an empirical quantitative, qualitative, or mixed-methods design.
2. Examined at least one of the following outcomes: PTSS, anxiety, depression, problematic substance use, or behavioral addictions.
3. Were conducted among Israeli populations, including the general population as well as relevant subgroups such as reservists, evacuees, bereaved individuals, adolescents, or children exposed to the war context.
4. Were published between October 7, 2023 and October 7, 2025.

Eligible designs included cross-sectional, longitudinal, cohort, and case-control studies. Qualitative and mixed-methods studies were included when they reported empirical data relevant to the review objectives. Commentaries, editorials, theoretical papers, case reports, dissertations, and other non-empirical publications were excluded. Only studies published in English or Hebrew were included. Studies were excluded if they did not assess at least one of the predefined symptom-domains (PTSS, depression, anxiety, or addiction-related outcomes).

### Information sources and search strategy

A systematic search was conducted in *PubMed, PsycINFO, Scopus*, and *Web of Science*. The initial search was performed on July 27, 2025 and updated through December 30, 2025, which served as the final search date. Reference lists of included studies were screened to identify additional eligible publications.

Search strategies combined controlled vocabulary (e.g., MeSH terms) and free-text keywords relating to:

1. Exposure to the October 7 attacks, the ongoing war, terror, or related conflict terms;
2. Psychiatric outcomes, including PTSS/PTSD, anxiety, depression, substance use, and behavioral addictions; and
3. Psychiatric responses at the individual or population level. The complete search strategy for each database, including all search terms and Boolean operators, is provided in Appendix S1. Searches were limited to publications from October 2023 onward and to articles published in English or Hebrew.

### Selection process

All records were exported and managed using a structured screening tool (Google Sheets). Duplicate records were manually removed prior to screening.

Titles and abstracts were independently screened by two reviewers. Full texts of potentially eligible studies were retrieved and independently assessed for inclusion by the same reviewers. Disagreements were resolved through discussion and, when necessary, consultation with a third reviewer. Reasons for exclusion at the full-text stage were documented. The selection process is summarized in the PRISMA flow diagram.

### Data collection process

Data were independently extracted by two reviewers using a standardized extraction form. Extracted data were cross-checked, and discrepancies were resolved through discussion and consensus. In two instances where full-text articles were not accessible through institutional databases, corresponding authors were contacted directly. The authors provided the full manuscripts, which were subsequently assessed for eligibility. Exposure was not a focal outcome of this review and was not coded or rated by the review team. Where the primary studies characterized exposure (e.g., as direct, indirect, cumulative, or role-related), these characterizations were recorded as reported by the original authors. Accordingly, the exposure categories referred to in the synthesis reflect the descriptions used in the primary studies rather than an *a priori* or independently coded exposure framework, and they are presented descriptively alongside the symptom-domain findings.

### Data items

For each included study, the following information was extracted:

- Authors and year of publication.
- Study design.
- Population characteristics (including age and gender distribution) when available.
- Population subgroup (e.g., general population, reservists, evacuees, bereaved individuals, youth).
- Timing of assessment relative to October 7, 2023.
- Exposure characteristics, including direct, indirect, cumulative and role-related exposure when available.
- Psychiatric outcomes assessed.
- Measurement instruments used.
- Key quantitative indicators (e.g., prevalence rates, mean scores, effect sizes) or qualitative findings.
- Functional outcomes (e.g., quality of life, occupational functioning), when reported.

### Risk of bias assessment

Risk of bias was assessed independently by two reviewers using design-specific appraisal tools. Cohort studies were evaluated using the Joanna Briggs Institute (JBI) Critical Appraisal Checklist for Cohort Studies, cross-sectional studies were assessed using the JBI Critical Appraisal Checklist for Analytical Cross-Sectional Studies, and qualitative studies were evaluated using the JBI Critical Appraisal Checklist for Qualitative Research (18). Risk of bias ratings were summarized descriptively and informed the interpretation of findings (Appendix S2).

Each study was appraised across relevant methodological domains, including sampling strategy, measurement validity, confounding control (where applicable), and appropriateness of analytic methods. Disagreements were resolved through discussion and consensus.

No studies were excluded based on quality assessment alone. Instead, methodological limitations were considered qualitatively in the interpretation of findings.

### Synthesis of results

Given substantial heterogeneity in study designs, populations, exposure definitions, outcome measures, and timing of assessments, a meta-analysis was not conducted. This heterogeneity precluded meaningful quantitative pooling. Specifically, studies varied in the operationalization of exposure, in outcome instruments and screening cutoffs (e.g., brief two-item screeners versus full symptom inventories), and in the timing of assessment relative to October 7, and in their sampling frames. In addition, substantial sample overlap across reports drawn from shared national panels would violate the assumption of independent samples required for meta-analytic pooling. Findings were synthesized narratively and summarized in structured tables.

Studies were grouped by outcome domain (PTSS, anxiety, depression, addiction) and examined in relation to exposure characteristics and population subgroup where available. Patterns, consistencies, and discrepancies across studies were identified, with attention to methodological limitations and contextual factors.

Given the conceptual and empirical overlap between anxiety and depressive symptoms, and variability in their operationalization, findings were synthesized jointly, with distinctions noted where relevant.

## Results

**Figure 1:**
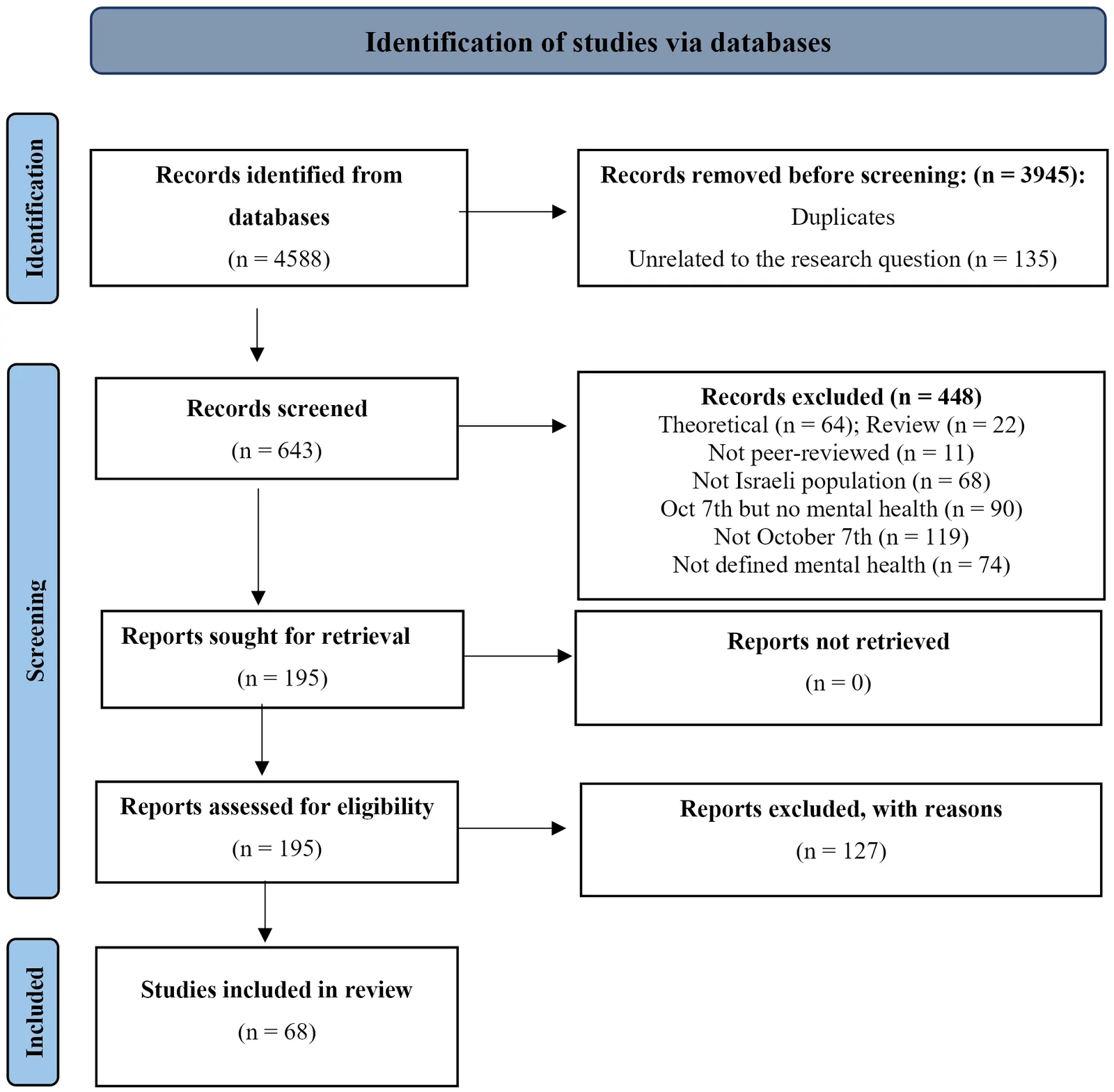
PRISMA 2020 flow diagram of the study selection process. Note. Duplicated records identified across databases were removed before screening. Records unrelated to the research question (e.g., references to events prior to 2023) were removed during a preliminary relevance check. Two reports initially inaccessible through institutional databases were obtained from the corresponding authors; no reports remained unretrieved. The 127 reports excluded at the full-text stage did not meet the predefined eligibility criteria (e.g., non-Israeli population, or no assessment of a predefined symptom domain).

The database searches identified 4,588 records. A total of 3,945 duplicate or clearly irrelevant records were removed prior to title and abstract screening. The remaining 643 records were screened, of which 448 were excluded, leaving 195 full-text articles were assessed for eligibility. Of these, 127 were excluded for documented reasons, and 68 studies met the inclusion criteria and were included in the final synthesis. Reasons for exclusion at the full-text stage are detailed in the PRISMA flow diagram (**Figure 1**).

The 68 included studies were published between October 7, 2023, and October 7, 2025, and encompassed cross-sectional, longitudinal, or repeated-measures designs. Study populations included general adult samples as well as high-risk or role-exposed groups, such as reservists, evacuees, bereaved individuals, and war-exposed children. A detailed overview of study characteristics, populations, outcomes assessed, and measurement instruments is presented in **Table 1**.

**Table 1:** Characteristics of included studies.

Study ID		Population									
Authors (year)	Study design	Sample size (total N)	Source of participants	Population subgroup	Age (M)	Female (%)	Timing of assessment since October 7 (in months)	Mental health (depression, anxiety, PTSD, addictions)	Measures	Key findings	Risk of bias
Aloni et al. (19)	Cohort	145	Patients followed at the Multiple Sclerosis Center, Sheba Medical Center	Adults with relapsing–remitting multiple sclerosis (pwMS)	39.9	68.3	6-8 post	Depression Anxiety	CES-D STAI PACT	Anxiety and depression were not associated with relapse frequency among adults with multiple sclerosis.*	Moderate
Aloush et al. (20)	Cross sectional	246	Convenience sampling via outpatient clinics, patient associations and online fibromyalgia groups	Adults with diagnosed fibromyalgia	48.7	89.4	3 post	Depression Anxiety	HADS PHQ-9 GAD-7	Increased anxiety and depression were associated with treatment disruption, pain, and somatic symptoms among adults with fibromyalgia.*	Moderate
Al-Said & Braun-Lewensohn (21)	Cross sectional	762	NR	Bedouin teenagers	16.4	68.4	3-5 post	Anxiety	STAI-S	Greater subjective exposure was associated with higher anxiety among Bedouin adolescents, particularly girls; sense of coherence was protective.*	Low
Amsalem et al. (22)	Cohort	(T1)=1052 (T2)=792 (T3)=689	Online panel (Panel4All)	Adult residing in northern and southern Israeli conflict zones	30.4	54	(T1) 4 post (T2) 5 post (T3) 7 post	Depression Anxiety PTSD	PHQ-9 PC-PTSD-5 GAD-7	Traumatic loss, displacement, and economic hardship were associated with persistently higher anxiety, depression, and PTSD among civilians living in conflict zones. At T1, T2, T3 respectively; 44%, 40%, and 37% (T3) met PC-PTSD-5 criteria for PTSD; 36%, 33%, and 31% (T3) met PHQ-9 criteria for depression; 32%, 29%, and 24% met GAD-7 criteria for anxiety.	Low
Amsalem et al. (23)	Cohort	(T1)=1052 (T2)=792 (T3)=689	Online panel (Panel4All)	Civilians living in northern and southern Israeli conflict zones	30.4	54	(T1) 4 post (T2) 5 post (T3) 7 post	Depression Anxiety PTSD	PHQ-9 PC-PTSD-5 GAD-7	Perceived betrayal was associated with persistently higher anxiety, depression, and PTSD, particularly among women.*	Low
Barel et al. (24)	Cohort	(T1)=512 (T2)=250	Online panel (iPanel)	Israeli adults	41.7	50.4	(T1) 1 pre (T2) 5 post	Depression Anxiety PTSD	DASS-21 ITQ	Higher self-coldness was associated with increased depression, anxiety, and posttraumatic symptoms among Israeli Jewish adults, whereas self-compassion and social support were protective. 27.2% met ITQ criteria for PTSD.	Low
Ben-David et al. (25)	Cross sectional	553	Online panel (Midgam)	Israeli Jewish adults	57.51	48.3	6 post	Anxiety PTSD	GAD-7 ITQ	Five days post-attack, high rates of peritraumatic distress and anxiety were reported among Israeli Jewish adults. 19.2% met GAD-7 criteria for anxiety and 41.1% met ITQ criteria for PTSD.	Low
Ben-Ezra et al. (26)	Cross sectional	2028	Online panel (iPanel)	Israeli Jewish adults	41.75	49.90	1 post	PTSD	ITQ	Acute PTSD and CPTSD were common following October 7 among Israeli Jewish adults, with CPTSD associated with greater war-related perceptual symptoms. 20.6% met ITQ criteria for PTSD and 14.7% met criteria for CPTSD.	Low
Bergman et al. (27)	Cross sectional	554	Convenience and snowball sampling via online platforms	Community older Israeli adults	73.9	58.8	3-5 post	Depression Anxiety PTSD	ITQ CES-D GAD-7	High rates of depression, PTSD, and anxiety were observed among older adults, with death anxiety identified as a key risk factor. 19.3% met ITQ criteria for PTSD; 48.4% met CES-D criteria for depression; 15.7% met GAD-7 criteria for anxiety.	Low
Boaz et al. (28)	Cross sectional	534	Representative random sample recruited online via survey company (Sekernet)	Israeli Jewish adults	NR	52.2	NR	Anxiety	GAD-7	Food insecurity during the war was associated with higher anxiety among Israeli civilians. 39.7% met GAD-7 criteria for anxiety.	Low
Dahan et al. (29)	Cross sectional	147	Lev Hasharon Mental Health Centre employees	Mental health workers	46.7	70.1	1 post	Anxiety	GAD-7	Higher anxiety among mental health workers was associated with prior trauma and media-related secondary exposure; resilience and religiosity were protective.*	Low
Eliashar et al. (30)	Cross sectional	968	Online panel (iPanel)	Israeli Jewish adults	41.5	50.6	1 post	Addiction (Substance use)	Self-report increased substance use	Increased substance use was associated with direct, indirect, and media-related exposure among Israeli adults, partly mediated by psychological distress.*	Low
Enav et al. (31)	Cross sectional	517	Online panel (iPanel)	Israeli adults	40.5	50.3	1 post	Anxiety PTSD	GAD-7 PCL-5	Greater war-related media exposure was associated with higher anxiety and posttraumatic stress among Israeli adults, moderated by emotion regulation strategies.*	Low
Enav et al. (32)	Cross sectional	517	Online panel (iPanel)	Israeli adults	40.5	50.3	2 post	Anxiety PTSD	GAD-7 PCL-5	Subjective but not objective exposure was associated with higher anxiety and posttraumatic stress among Israeli adults; emotion suppression increased risk.*	Low
Erdinast et al. (33)	Cohort	(T1)=118 (T2)=85	Recruitment via email lists and social media; follow-up of an existing research cohort	Young adults with ADHD vs. controls without ADHD	24.1	81.2	(T1) 4-21 pre (T2) 1 post	Anxiety	STAI	Anxiety increased following October 7 in both groups and declined over time; ADHD symptoms increased only among controls, whereas participants with ADHD showed no increase and slight symptom improvement.*	Moderate
Eshel et al. (34)	Cross sectional	1,608	Online internet panel (stratified, nationally representative Jewish Israeli sample)	Israeli Jewish adults	NR	50.4	.25 post	Depression Anxiety	BSI	Under extreme stress, Mizrahi individuals reported higher anxiety and depression than Ashkenazi individuals among Israeli adults.*	Moderate
Feingold et al. (35)	Cross sectional	415	Convenience and snowball sampling via online platforms	Jewish and Arab Israeli adults; predominantly residents of southern Israel	42.22	84.8	1 post	PTSD substance use/addiction	PCL-5 self-report change substances use	Increased post-attack use of tobacco, alcohol, tranquilizers, pain medications and sleep medications was reported among Israeli adults, with higher cannabis use among men. 31.4% met PCL-5 criteria for PTSD.	Low
Gilboa Schechtman et al. (36)	Cohort	(T1)=858 (T2)=509	Online panel (Panel4All)	Israeli Jewish adults	37.21	56.4	(T1) 3 Post (T2) 7–8 Post	Depression Anxiety PTSD	PHQ-8 GAD-7 PCL-5	High rates of probable PTSD, anxiety, and depression were observed three months post-attack, particularly among women. 32.2% met PCL-5 criteria for PTSD, 27.6% met PHQ-8 criteria for depression, and 18.8% met GAD-7 criteria for anxiety.	Low
Groweiss et al. (2)	Cohort	(T1)=908 (T2)=710	Online panel (Panel4All)	Ethnic groups: Jewish majority vs. Arab minority (Muslim Arabs)	41.01	50.70	(T1) 1.5 pre (T2) 1.5 post	Depression Anxiety PTSD	PHQ-9 GAD-2 ITQ	Post-attack increases in PTSD and depression, but not anxiety, were greater among Arab Israelis than among Jewish Israelis. PTSD increased from.*	Low
Haim-Nachum et al. (37)	Cross sectional	1,052	Online panel (Panel4All)	Civilians living in conflict zones (southern & northern Israel)	30.4	53.8	4 Post	Depression Anxiety PTSD	PHQ-9 GAD-7 PC-PTSD-5+K11	Exposure to morally injurious events was associated with high rates of depression, PTSD, and anxiety among Israeli civilians, partly mediated by treatment stigma. 44.2% met PC-PTSD-5 criteria for PTSD; 35.6% met PHQ-9 criteria for depression and 31.6% met GAD-7 criteria for at least moderate anxiety.	Low
Hamama et al. (38)	Cross sectional	366	Online panel (iPanel)	Evacuees vs. non-evacuees (controls) from southern & northern Israel	41	70	7 Post	Depression Anxiety	PHQ-2 GAD-2	Compared with non-evacuees, evacuees reported higher anxiety, depression, and traumatic life events, alongside lower physical health-related quality of life.*	Low
Hamama-Raz et al. (39)	Cross sectional	2,028	Online panel (iPanel)	Israeli adults with lifetime bereavement (with or without additional October 7-related loss)	NR	NR	1 Post	PTSD	ITQ	Latent high-grief profiles were associated with markedly increased risk of PTSD and CPTSD, with negative cognitive emotion regulation also predicting membership in high-grief classes.*	Low
Hazan-Liran & Karni-Vizer (40)	Cross sectional	126	Convenience and snowball sampling via online platforms	Evacuees from Northern Israel; employed and unemployed adults	29.88	67.5	5-8 Post	Anxiety	GAD-7	Unemployed evacuees reported higher anxiety than employed evacuees, suggesting employment served as a protective factor.*	Low
Klapper-Goldstein et al. (41)	Cross sectional	502	Women delivering at Soroka University Medical Center (tertiary hospital)	Postpartum women (Jewish and Bedouin) delivering singleton live births	29.3	100	36-48 Pre During-1 Post	Depression Anxiety	EPDS STAI PBQ	Delivery during the war was associated with increased postpartum depression and anxiety among pregnant women. 26.6% (delivered during the war) and 12.4% (comparison) met EPDS criteria for depression; 34.3% and 17.0% met STAI criteria for anxiety.	Low
Laufer (42)	Cross sectional	161	Israeli Defense Forces reserve units involved in body handling after Oct 7	Reserve soldiers involved in body handling and identification of human remains	41.17	8.1	2-3 Post	PTSD	PCL-5	Approximately 20% met criteria for PTSD among Israeli adults, with higher risk under greater task difficulty and lower well-being. 20% met PCL-5 criteria for PTSD.	High
Levi-Belz et al. (43)	Cohort	(T1) NR (T2) NR (T3) NR (T4) 614	Online panel (Panel4All)	Bereaved vs. non-bereaved adults (loss of close family member or close friend due to Oct 7 attack or subsequent war)	41.02	50.4	(T1) 1.5 Pre (T2) 1.5 Post (T3) 4.5 Post (T4) 8.5 Post.	Depression Anxiety PTSD	PHQ-2 GAD-2 ITQ	Bereavement following October 7 was associated with persistently higher PTSD, depression, and anxiety among Israeli civilians. 29.5% (bereaved) and 19.2% (non-bereaved) met ITQ criteria for PTSD; 37.3% and 22.4% met PHQ-2 criteria for depression; 37.3% and 21.5% met GAD-2 criteria for anxiety.	Low
Levi-Belz et al. (44)	Cohort	(T1) 908 (T2) 710 (T3) 614	Online panel (Panel4All)	Parents of minor children (subset of general population)	41.02	50.3	(T1) 1.5 Pre (T2) 1.5 Post (T3) 12 Post	Depression Anxiety PTSD	PHQ-2 GAD-2 ITQ	Reserve-duty combatants and bereaved individuals showed the highest and most persistent psychological distress among Israeli adults.*	Low
Levi-Belz et al. (45)	Cohort	(T1) NR (T2) NR (T3) 634	Online panel (Panel4All)	General adult population; subgroup of directly exposed individuals (present in Gaza envelope communities during the attack)	40.89	50.5	(T1) 1.5 Pre (T2) 1.5 Post (T3) 4.5 Post	Depression PTSD	PHQ-2 ITQ	Direct exposure was associated with higher and more persistent PTSD and depression among Israeli civilians. 40.3% met ITQ criteria for PTSD; 45.2% met PHQ-2 criteria for depression.	Low
Levi-Belz et al. (46)	Cohort	(T1) 908 (T2) 710 (T3) 600	Online panel (Panel4All)	Israeli Jewish adults	41.02	50.3	(T1) 1.5 Pre (T2) 1.5 Post (T3) 12 Post	Depression Anxiety PTSD	PHQ-2 GAD-2 ITQ	PTSD-depression comorbidity post-attack predicted the highest long-term symptom burden among Israeli adults.*	Low
Levi-Belz et al. (47)	Cohort	(T1) 908 (T2) 710	Online panel (Panel4All)	Israeli adults (Jewish and Arab general population)	41.01	51.1	(T1) 1.5 pre (T2) 1.5 post	Depression Anxiety PTSD	PHQ-2 GAD-2 ITQ	Female sex was associated with higher odds of PTSD, depression, and anxiety among Israeli adults. 34.5% (females) and 24.9% (males) met ITQ criteria for PTSD; 56.6% (females) and 32.6% (males) met PHQ-2 criteria for depression; 55.1% (females) and 29.4% (males) met GAD-2 criteria for anxiety	Low
Levi-Belz et al. (3)	Cohort	(T1) 908 (T2) 710	Online panel (Panel4All)	Israeli Jewish adults	41.01	51.1	(T1) 1.5 pre (T2) 1.5 post	PTSD	ITQ	Loneliness moderated the association between posttraumatic stress symptoms and suicidal ideation among Israeli adults. 16.2% (T1) and 29.8% (T2) met ITQ criteria for PTSD	Low
Levi-Belz et al. (4)	Cohort	(T1) 908 (T2) 710	Online panel (Panel4All)	Israeli Jewish adults	41.01	51.1	(T1) 1.5 pre (T2) 1.5 post	Depression PTSD	PHQ-2 ITQ	Post-attack betrayal increased the likelihood of probable PTSD and depression among Israeli adults. **1**6.2% (T1) and 29.8% (T2) met ITQ criteria for PTSD, 31.3% (T1) and 44.8% (T2) met PHQ-2 criteria for probable depression.	Low
Levi-Belz et al. (5)	Cohort	(T1) 908 (T2) 710	Online panel (Panel4All)	Israeli adults (Jewish and Arab general population)	41.01	51.10%	(T1) 1.5 pre (T2) 1.5 post	Depression Anxiety PTSD	PHQ-2 GAD-2 ITQ	Post-attack prevalence of PTSD, anxiety, and depression increased markedly, particularly among women. 16.2% (T1) and 29.8% (T2) met ITQ criteria for PTSD; 31.3% and 44.8% met PHQ-2 criteria for depression; 24.9% and 42.7% met GAD-2 criteria for anxiety.	Low
Levi-Belz et al. (48)	Cohort	(T1) 908 (T2) 710	Online panel (Panel4All)	Israeli Jewish adults	41.01	51.1	(T1) 1.5 pre (T2) 1.5 post	Depression	PHQ-2	Post-attack depression was associated with increased suicidal ideation among Israeli adults, moderated by low belongingness. 31.3% (T1) and 44.8% (T2) met PHQ-2 criteria for depression.	Low
Levin et al. (49)	Cohort	(T1) 4035 (T2) 2,659 (T3) 4,002 (T4) 2,768	Online panel (iPanel)	Israeli Jewish adults	NR	47.9	(T1) 69 pre (T2) 18 pre (T3) 2 post (T4) 5 post	PTSD	PCL-5	Trauma vulnerability and chronic PTSD trajectories were associated with higher exposure and prior trauma among Israeli adults.*	Low
Lifshin et al. (50)	Cohort	(T1) 2,659 (T2) 4,002 (T3) 2,768 (T4) 2757	Online panel (iPanel)	Israeli Jewish adults	45.67	49.4	(T1) 7 pre (T2) 2 post (T3) 5 post (T4) 8 post	Addiction (gambling)	PGSI	Increased gambling during the war was observed among men without prior gambling problems, particularly those with high emotion regulation difficulties. 14.7% met PGSI criteria for gambling (low-risk or above).	Moderate
Lipskaya-Velikovsky eal. (6)	Cross sectional	863	Convenience and snowball sampling via online platforms	Higher education students	NR	81%	1 Post	Depression Anxiety	DASS-21	Higher anxiety and depression were associated with lower life satisfaction and resilience among Israeli adults.*	Low
Mayer et al. (51)	Cross sectional	517	Online panel (iPanel)	Arab and Jewish Israeli adults (representative sample)	41.42	50.3	1 Post	Anxiety PTSD	GAD-7 PCL-5	PTSD rates were similar among Jewish and Arab Israeli adults, despite differing exposure levels.*	Low
Maytles et al. (52)	Cross sectional	118	Organizations caring for Holocaust survivors + community-dwelling older adults	Holocaust Survivors with high PTSD symptom, and with low PTSD symptom levels and a comparison group	85.71	50	2–3 Post	Depression Anxiety PTSD	PHQ-4 ICD-11 PTSD	Holocaust survivors with pre-existing PTSD reported greater post-attack anxiety, depression, and PTSD compared with non-survivors.*	Low
Mor et al. (53)	Cross sectional	400	Sekernet online survey platform (national panel, stratified sampling)	Jewish mothers up to two years postpartum, without prior diagnosed mental illness	30.85	100	3 Post	Anxiety PTSD	GAD-7 PCL-5	Higher exposure and media use were associated with greater anxiety and distress among postpartum mothers.*	Low
Palgi et al. (54)	Cross sectional	375	Convenience and snowball sampling via online platforms	general population	48.98	80.1	2 Post	Depression Anxiety PTSD	SCES-D GAD-7 ITQ STO	PTSD and subjective traumatic outlook independently predicted higher anxiety and depression among Israeli adults. 35.7% met ITQ criteria for PTSD, 15.1% met SCES-D criteria for depression, and 57.4% met GAD-7 criteria for anxiety.	Low
Palgi et al. (55)	Cohort	(T1) 399 (T2) 297	Online panel (iPanel)	Older adult Yom Kippur War veterans (Jewish males)	73.25	0	(T1) 6 Pre (T2) 1 post	Depression PTSD	PHQ-9 ITQ	Pre-war despair predicted post-attack PTSD and depression among older adult veterans.*	Low
Peleg et al. (56)	Cross sectional	690	Convenience and snowball sampling via online platforms	Israeli adults; evacuees (35.4%) and non-evacuees	41.38	75.5	4 Post	Depression Anxiety PTSD	PHQ-2 GAD-2 PCL-5	High levels of anxiety, depression, and PTSD were observed in the general Israeli population during the war. 33.5% met PCL-5 criteria for PTSD; 47.4% met PHQ-2 criteria for depression; 50.0% met GAD-2 criteria for anxiety.	Moderate
Pitcho et al. (57)	Cross sectional	445	Convenience and snowball sampling via online platforms	Hebrew-speaking Israeli adults aged 18–67	30.9	68.5	During the Israel–Hamas war following October 7, 2023 (exact dates NR)	Anxiety	GAD-2	Anxiety was associated with emotional eating and poorer body image, particularly among women. 35% met GAD-2 criteria for anxiety.	Low
Rachamim et al. (58)	Cross sectional	220 (dyads)	parents that approach for their treatment to their child to Oti Association’s Cohen-Harris Resilience Center.	dyads of mothers and children between ages 3 to 12	7.70 (children) 39.41 (parents)	55.5 (children) 100 (parents)	1-9 Post	PTSD	YCPC CPSS-5 PDS-5 CTS	High rates of PTSD were observed among treatment-seeking children, with maternal PTSD predicting symptoms in preschool-aged children. 69% of preschool children met YCPC criteria for PTSD, 49.2% of school-age children met CPSS-5 criteria for PTSD, and 32.4% of mothers met PDS-5 criteria for PTSD.	Low
Reznik et al. (59)	Cross sectional	1,071	Convenience and snowball sampling via online platforms	Ukrainian and Israeli adult women	38.3	100	1-5 Post	Depression Substance misuse	PHQ-9 Self-reported substance use	Israeli women reported higher fear of war and depression, while Ukrainian women experienced greater social loneliness, psycho-emotional deterioration, and increased substance misuse.*	Moderate
Ring et al. (60)	Cross sectional	175	Social media groups for pregnant women	Pregnant women	31.14	100	1 Post	PTSD	PCL-5	Among pregnant women, higher post-traumatic stress and war-related concerns were significantly associated with lower self-mastery and greater intolerance of uncertainty.*	Low
Rozenblat et al. (61)	Cross sectional	92	Online recruitment via social media, Autism Child and Family Lab (HUJI), Azrieli National Centre for Autism, ALUT	Autistic children (ages 3–17) and their parents; comparison group of non-autistic children and parents	39.8	75.4	1 Post	Depression Anxiety PTSD	DASS-21 CATS	Clinically significant post-traumatic stress symptoms were observed among children after October 7, with greater severity among autistic children and increased depression and anxiety among their parents.*	Low
Rubinstein et al. (62)	Cross sectional	1511	Online panel (Midgam)	Israeli Jewish adults	46.9	52.5	2 Post	PTSD	PCL-5-20	Trauma-related clinical symptoms were centrally connected to dissociation, while playfulness was associated with lower trauma-related symptomatology.*	Low
Saar-Ashkenazy et al. (63)	Cross sectional	286	Convenience and snowball sampling via online platforms	Jewish Israeli adults living in southern Israel; loss vs. no-loss groups	39.51	71.3	1-1.5 Post	PTSD	PCL-5	Problem-focused coping was associated with higher resilience, while emotion-focused coping linked to lower resilience; loss significantly reduced resilience only among individuals using low levels of both coping strategies.*	Low
Saar-Ashkenazy et al. (64)	Cross sectional	374	Convenience and snowball sampling via online platforms	Adoult living in Southern Israel. FRs (i.e. firefighters, paramedics, police force members, and active military service; n = 77), non-FRs (n = 297)	39.24	50.8	1 Post	Traumatic stress symptoms (TSS)	PCL-5	Traumatic stress symptoms (TSS) were negatively associated with resilience among first responders and civilians, but this association was not significant among first responders actively engaged in assistance.*	Low
Saar-Ashkenazy et al. (65)	Cross sectional	295	Online survey among residents of southern Israel	First responders (police, firefighters, paramedics, emergency medical staff, security forces) and civilian comparison group	39.05	50.8	1 Post	PTSD	PCL-5	Higher traumatic stress symptomatology was associated with lower psychological flexibility, particularly among first responders.*	Moderate
Sarid et al. (66)	Cross sectional	366	Online panel (iPanel)	Evacuees (north & south) and non-evacuated Israeli adults	40.93	62.8	7 Post	Depression Anxiety	PHQ-4	Traumatic life events were associated with higher anxiety and depression among both evacuees and non-evacuees.*	Low
Sason et al. (67)	Cross sectional	297	Single methadone maintenance treatment (MMT) clinic, Tel Aviv Sourasky Medical Center	Adults with opioid use disorder receiving MMT	56	21.9	1-2 Post	Anxiety	GAD-7	High anxiety levels were common among methadone maintenance treatment patients one month after October 7. 42.8% met GAD-7 criteria for anxiety.	Low
Sedoff et al. (68)	Cross sectional	415	Convenience and snowball sampling via online platforms and Achva Academic College mailing lists	Israeli adults (majority Jewish; community sample)	NR	84.8	1 Post	PTSD	PCL-5	Lower perceived social support was associated with greater PTSD severity following October 7.*	Low
Shamai-Leshem et al. (69)	Cross sectional	73	Convenience sampling via professional therapist groups in Israel	Israeli psychotherapists treating trauma survivors	42.8	88	2-4 Post	PTSD	PCL-5	Probable PTSD was observed among psychotherapists not directly exposed to October 7. 18% met the PCL-5 criteria for PTSD.	Low
Shamoa-Nir (70)	Cross sectional	173	Convenience and snowball sampling via online platforms	Jewish Israeli adults from Northern, Southern, and Central Israel	28.36	50.9	1-2 Post	Depression Anxiety	DASS-21	Direct exposure to the October 7 attacks was associated with higher anxiety and depression.*	Low
Shapira et al. (71).	Repeated cross sectional	(W1) 1216 (W2) 915	Convenience and snowball sampling via online platforms	Young adults (18–24)	20.8	67.6	5-24 Pre 3-5 Post	PTSD	PCL-5	PTSD rates and severity increased among young adults after October 7. 43.3% met PCL-5 criteria for PTSD.	Low
Shechory-Bitton et al. (72)	Cross sectional	483	Online panel (iPanel)	Evacuee and non- evacuee parents of children under 18	39.67	NR	3 Post	PTSD	PCL-5	Evacuated parents reported higher post-traumatic stress, and higher parental PTS was associated with greater child aggression and social problems.*	Low
Shelef et al. (73)	Cross sectional	806	IDF Combat Stress Reaction Unit (CSRU)	Israeli reserve soldiers (combat & combat-support)	30.26	3.6	25-10 Post	PTSD	PCL-5	Probable PTSD was common among IDF reserve soldiers following participation in the October 7 war and was associated with intense combat-related exposures. 58.1% met PCL-5 criteria for PTSD.	Low
Shklarski et al. (74)	Cross sectional	223	Convenience sample of Israeli therapists recruited via email and online invitation	Mental health therapists treating populations affected by the October 7 attack (bereaved families, hostage families, evacuees, reserve soldiers, survivors)	NR	87.4	4-6 Post	Depression Anxiety	DASS-21	High levels of secondary traumatic stress were common among Israeli therapists treating war-affected populations following October 7.*	Low
Shmulewitz et al. (75)	Cross sectional	3,998	Online panel (iPanel)	Jewish Israeli adults (18–70)	41.4	50.5	2 Post	PTSD	PCL-5	Greater exposure to online hate speech was associated with higher PTSD symptoms following the October 7 attacks. 25% met PCL-5 criteria for PTSD	Low
Shrira et al. (76)	Cohort	(T1) 1071 (T2) 582	Online panel (iPanel)	Holocaust descendants (children & grandchildren) vs. comparison descendants (Israeli Jews without Holocaust family background)	44.8	47.9	(T1) 12 Pre (T2) 2 Post	PTSD	ITQ	Probable PTSD rates increased primarily among Holocaust descendants during the war. 10.6% met ITQ criteria for PTSD	Low
Shrira et al. (7)	Cohort	(T1) 399 (T2) 297	Online panel (iPanel)	Yom Kippur War veterans (older adult male veterans)	73.25	0	(T1) 5 Pre (T2) 2 Post	Depression Anxiety PTSD	PHQ-9 GAD-7 ITQ	Probable PTSD increased among Yom Kippur War veterans after October 7. 5.4% (T1) and 13.1% (T2) met ITQ criteria for PTSD	Low
Shrira & Palgi (77)	Cross sectional	428	Convenience and snowball sampling via online platforms	Israeli Jewish adults	48.36	80.7	1–2 Post	PTSD	ITQ	Young adults showed higher rates of acute stress symptoms and probable PTSD than older adults; 42.8% (young adults) and 13.7% (older adults) met ITQ criteria for PTSD	High
Sowan et al. (78)	Cohort	(T1) 393 (T2) 338	Survey company panel; web-based recruitment with oversampling of Arab citizens	Israeli civilians with indirect war exposure (second & third war circles; Jewish and Arab citizens)	42.01	47	(T1) 1 Post (T2) 6 Post	PTSD	PCL-5	War exposure predicted higher posttraumatic stress symptoms at six months only among individuals with medium or high levels of demoralization shortly after the war onset.*	Low
Walter et al. (79)	Cross sectional	194	Convenience and snowball sampling via online platforms	Palestinian Arab Minority in Israel; emerging adults (students)	30.8	87	3 Pre and up to 4 Post	Depression Anxiety	PHQ-4	Among Palestinian emerging adults in Israel, higher past and future temporal focus were associated with greater anxiety and depression, while the outbreak of war did not significantly impact these mental health indicators.*	Low
Zerach (80)	Cross sectional	293	Convenience and snowball sampling via online platforms	Wives of Israeli reserve soldiers	36	100	6-8 Post	Depression Anxiety	PHQ-9 GAD-7	Spouses of reserve soldiers with high perceived stress reported higher depression and anxiety.43% met PHQ-9 criteria for depression; 25.9% met GAD-7 criteria for anxiety	Low

*S*CES-D, Center for Epidemiologic Studies Depression Scale; STAI/STAI-S, State–Trait Anxiety Inventory; PACT, Perceived Ability to Cope with Trauma; HADS, Hospital Anxiety and Depression Scale; PHQ-9/PHQ-8/PHQ-4/PHQ-2, Patient Health Questionnaire; GAD-7/GAD-2, Generalized Anxiety Disorder scale; CTS - Continuous Traumatic Stress; PC-PTSD-5, Primary Care PTSD Screen for DSM-5; PCL-5/PCL-6/PCL-5-20, PTSD Checklist for DSM-5; STO, Subjective traumatic outlook; ITQ, International Trauma Questionnaire; DASS-21, Depression Anxiety Stress Scales; BSI, Brief Symptom Inventory; EPDS, Edinburgh Postnatal Depression Scale; PBQ, Postpartum Bonding Questionnaire; PGSI, Problem Gambling Severity Index; CATS, Child and Adolescent Trauma Screen; CPSS-5, Child PTSD Symptom Scale for DSM-5; PDS-5, PTSD Diagnostic Scale for DSM-5; ICD-11 PTSD, Posttraumatic Stress Disorder according to ICD-11 criteria; YCPC, Young Child PTSD Checklist. Quantitative indicators in the Key findings column report the proportion of participants meeting each instrument’s threshold for a clinically significant outcome. “Meeting criteria” was defined per instrument as follows: PCL-5 ≥31–33 (probable PTSD, depending on the cutoff used in the original study); PC-PTSD-5 ≥3; CPSS-5 ≥31; ITQ by the ICD-11 diagnostic algorithm; PHQ-2 ≥3; GAD-2 ≥3; PHQ-9 ≥10 and GAD-7 ≥10 (at least moderate symptoms); EPDS ≥10; STAI >39; DASS-21 by published subscale norms; PGSI ≥1 (low-risk-or-above gambling). When studies reported severity-band distributions rather than a single proportion, the proportion meeting criteria was derived by summing the categories meeting the predefined threshold for each instrument. An asterisk (*) indicates that no proportion meeting criteria could be derived, either because the study reported only continuous scores (means, medians, or effect estimates) or because it did not specify the threshold applied. Risk of bias was independently appraised using the JBI critical appraisal checklists for cross-sectional and cohort studies; each study’s overall rating (Low/Moderate/High) reflects the number of items rated “yes,” with full item-level ratings and rating thresholds provided in the Appendix.

Risk of bias was assessed using design-specific JBI appraisal tools. Common methodological limitations included non-probability sampling, reliance on self-report measures, and the predominance of cross-sectional designs, limiting causal inference. A summary of the risk of bias assessment is provided in Appendix S2.

### Posttraumatic Stress Symptoms

Posttraumatic stress symptoms (PTSS) were examined in 45 studies of the 68 included studies, including 36 population-based studies and 9 conducted with high-risk or duty-related groups. Of these, 18 studies employed longitudinal or repeated-measures designs. Elevated PTSS levels and above-cutoff rates for probable PTSD were consistently reported following October 7. Above established clinical screening thresholds were documented in the general adult population during the initial months of the war (35, 54, 56). Rates were particularly high among treatment-seeking and role-exposed groups, including IDF reservists seeking care (73), individuals who lost a close family member or friend during the October 7 attack or the subsequent war (43), and young adults (71, 77). Longitudinal finding indicated heterogeneous symptom trajectories over time. While PTSS declined over time in some populations, symptoms remained elevated among subgroups characterized by sustained exposure or role-related strain (43–45, 49).

PTSS severity varied systematically according to types of trauma exposure. In reservist samples, specific combat-related events, such as hand-to-hand combat, exposure to human remains, and physical injury, were associated with higher PTSS and distinct symptom clusters (73). Elevated PTSS was also linked to sustained threat contexts and cumulative or indirect stressors, including prolonged missile attacks, broader war-related exposure, and role-linked burdens such as body-handling tasks (42, 70, 78).

Beyond exposure characteristics, PTSS severity was also associated with psychosocial vulnerabilities and protective resources. Higher symptom levels were observed among individuals reporting maladaptive emotion-regulation strategies, reduced psychological resources, and elevated demoralization early in the war (39, 75, 78). In contrast, greater perceived social support, particularly from friends or significant others, was associated with lower PTSS severity (68).

PTSS vulnerability also varied according to prior trauma history and developmental context. Among older adults with previous war exposure, intrusive cognitive linkage of past and current traumatic events was associated with elevated PTSS and affective symptoms (77). Higher PTSS rates were also observed among descendants of Holocaust survivors, particularly when parental or grandparental PTSD was reported (7, 76). Among war-exposed children, maternal posttraumatic symptoms were strongly associated with child PTSS, especially in preschool-aged samples (58).

### Anxiety and depression

Anxiety and depressive symptoms were widely assessed in the reviewed studies and emerged as central affective responses in the months following October 7. Multiple population-based studies documented marked elevations relative to available pre-attack baselines, with substantial proportions exceeding established screening clinical cutoffs during the early phase of the war (5, 54, 56). Longitudinal and repeated-measures findings indicated that although symptom levels declined over time for some groups, anxiety and depressive symptoms remained elevated among individuals exposed to sustained threat or ongoing stressors, including reserve-duty participants, evacuees, and bereaved individuals (43–45). These patterns suggest that anxiety and depressive symptoms were particularly sensitive to prolonged stress exposure rather than discrete traumatic events alone.

Anxiety and depressive symptoms were systematically associated with both direct trauma exposure and broader contextual stressors. Direct exposure to traumatic events, loss of close others, forced displacement, income loss, and prolonged missile-attack contexts were each linked to elevated symptom severity (22, 38, 70). Indirect and cumulative stressors, including food insecurity and extensive media exposure, were also associated with increased anxiety and depressive symptoms, even after adjustment for sociodemographic characteristics and prior mental health status (28, 31, 32). In some studies, perceived stress mediated the association between war exposure and anxiety and depression (53, 70), underscoring the role of stress appraisal in persistence of anxiety and depressive symptoms.

Pronounced group differences were repeatedly observed. Women consistently reported higher anxiety and depressive symptom levels than men across samples (22, 36, 47). Elevated symptom levels were also documented among ethnic minority groups, including Arab Israelis and Mizrahi participants, (Jews of Middle Eastern and North African descent) particularly under conditions of high stress (2, 22, 34). Additional vulnerability was observed among individuals facing socioeconomic or role-related strain, such as unemployed evacuees, postpartum women delivering during the war, spouses of reserve soldiers, and caregivers of war-exposed children (40, 41, 61, 80).

Beyond structural factors, lower levels of personal resources (e.g., psychological capital, self-mastery, resilience) and maladaptive coping patterns (e.g., emotional suppression, self-coldness) were associated with greater symptom severity (21, 24, 27, 32, 40). Anxiety and depressive symptoms were also linked to reduced health-related quality of life and daily functioning, even when symptoms severity declined over time (6, 36, 38).

### Addiction-related outcomes

Addiction-related outcomes were examined in only three studies published during the two years following October 7, two of which focused on substance use. Both studies reported post-attack increases in substance use (30, 35). Increased use was associated with greater exposure severity, psychological distress, and prior mental health difficulties. Direct exposure emerged as the strongest correlate of increased substance use, while psychological distress partially mediated associations between indirect exposure and substance use. Differential risk patterns were observed by gender and exposure level, including higher cannabis use among men and greater tranquilizer use among individuals with higher exposure.

The single study examining behavioral addictions focused on gambling (50). Increased gambling was observed primarily among men without prior gambling problems who reported greater difficulties in emotion regulation. No comparable increase was observed among women, and lower emotion regulation difficulties appeared protective among individuals with higher baseline gambling risk. Overall, the limited evidence suggests selective, exposure- and distress-related increases in addiction-related outcomes rather than generalized population-wide escalation.

## Discussion

The synthesis of evidence indicates that the psychiatric impact of the October 7 attacks and the ensuing war did not manifest as a unitary deterioration across symptom domains, but as a differentiated pattern in which persistence, severity, and risk varied systematically across subgroups. Across PTSS, anxiety, depression, and more preliminarily, addiction-related outcomes, psychiatric burden was consistently associated with the types of trauma exposure, the continuity of threat, and pre-existing structural and personal vulnerabilities, rather than with exposure occurrence alone. At the same time, these domains did not operate in isolation. PTSS, anxiety, and depressive symptoms frequently co-occurred, consistent with the well-documented comorbidity among these conditions following mass trauma (81, 82). The domain-specific patterns described below therefore reflect differences in emphasis and trajectory across partly overlapping conditions, rather than mutually exclusive responses.

A first cross-cutting theme concerns the role of sustained and cumulative exposure. Across domains, persistent or elevated symptoms were more frequently observed among individuals exposed to continuous threat, cumulative stressors, or role-related strain, including reservists, bereaved individuals, evacuees, and those in high-demand roles (e.g., 2, 22, 23, 32, 37–40, 42, 56). This pattern is consistent with broader trauma research showing that PTSD risk varies not only by the occurrence of trauma exposure but also by exposure proximity, intensity, and cumulative direct and indirect trauma burden (83, 84). For PTSS, specific combat-related experiences, such as close-quarter combat, physical injury, and perceived responsibility for civilian harm, were associated with higher symptom levels and distinct symptom profiles (37, 42), aligning with theoretical models of the psychiatric impact of ethically charged or role-embedded experiences (85, 86). For anxiety and depression, symptom persistence appeared to reflect cumulative stress load, chronic uncertainty, and the erosion of coping resources rather than discrete traumatic events alone (e.g., 87, 88). Across domains, then, the continuity and accumulation of exposure, more than its initial occurrence, were associated with the course of psychiatric symptoms under prolonged collective threat.

A second theme concerns individual, developmental, and social moderators of vulnerability. Maladaptive emotion-regulation strategies (e.g., 31, 75) and early demoralization (e.g., 78) were associated with higher symptom severity, consistent with broader evidence linking dysregulated affective processing to symptom maintenance (89, 90), whereas perceived social support, particularly from close relationship partners, was consistently associated with lower symptom severity (e.g., 24, 68, 91). Developmental and intergenerational factors further structured risk: intrusive linkages between past and current war exposure were associated with more severe symptoms among older adults (27, 55, 77), and elevated symptoms among descendants of Holocaust survivors (52, 76) and among war-exposed children whose mothers reported elevated posttraumatic symptoms (58) point to the reactivation of trauma-related vulnerabilities across generations. Socioeconomic and role-related strain compounded these risks, with higher symptom levels among evacuees, unemployed individuals, ethnic-minority populations, and spouses of reserve soldiers (38, 40, 56). Beyond symptom levels, elevated anxiety and depression were also associated with impaired daily and occupational functioning (6), underscoring that psychiatric burden in this context extended beyond symptom counts to everyday role performance. Observed gender differences, with women reporting higher internalizing symptoms (22), align with broader epidemiological findings (92) but warrant caution, as self-report instruments may under-capture stress responses more common among men, including physiological reactivity and externalizing behaviors (93–95).

Against this cross-domain convergence, addiction-related outcomes followed a more selective, context-dependent pattern. Examined in only three studies, the available evidence suggests targeted rather than population-wide increases: post-attack increases in substance use were associated with greater exposure severity and higher psychological distress (30, 35), with direct exposure the strongest correlate and psychological distress partially mediating increased use among indirectly exposed individuals. The single study of gambling identified a gender-specific pattern, with increased gambling primarily among men reporting greater emotion-regulation difficulties (50). Although the current evidence does not permit conclusions regarding the prevalence of substance use or gambling disorders during a prolonged collective crisis, these associations echo the same cross-cutting moderators, exposure intensity, regulatory capacity, and gender, identified across the other domains.

### Practice and policy implications

These findings indicate that broad, population-level psychiatric responses are unlikely to be sufficient on their own; targeted, context-sensitive approaches are needed to identify and support groups at heightened risk. The convergence of elevated risk among reservists, bereaved individuals, evacuees, intergenerational trauma-affected families, and socioeconomically strained groups suggests that screening and service delivery should prioritize these populations rather than assume uniform need across the population. Because PTSS, anxiety, and depression frequently co-occurred, integrated services addressing comorbid presentations, rather than single-disorder pathways, may be particularly appropriate in this context. The association between distress and impaired functioning further suggests that services should target not only symptom reduction but also the restoration of daily and occupational functioning. These priorities are consistent with trauma-informed models of care developed in other contexts of mass and community trauma (96, 97), which emphasize accessible, stepped, and population-tailored responses under conditions of ongoing threat.

### Research implications

Several priorities follow for future research. First, sustained longitudinal follow-up extending beyond the acute crisis period will be essential to determine whether observed symptom elevations represent transient stress reactions or consolidation into chronic psychiatric conditions requiring ongoing clinical intervention. Second, future studies should incorporate standardized diagnostic instruments rather than brief screeners alone, and should routinely assess functional outcomes alongside symptom prevalence, given evidence that impairment and symptoms do not fully overlap. Third, exposure should be operationalized systematically and integrated into analytic models, so that the differential contributions of exposure type, intensity, and cumulative burden can be distinguished from pre-existing vulnerability. Addressing these gaps would strengthen causal inference and support the development of predictive models capable of identifying individuals at greatest risk under conditions of protracted conflict.

### Strengths and limitations

This review has several strengths. To our knowledge, it is the first systematic synthesis of psychiatric outcomes across multiple symptom domains during the first two years following the October 7 attacks, integrating a rapidly emerging and heterogeneous literature within a single framework. Its domain-spanning scope allowed patterns of convergence and divergence across PTSS, anxiety, depression, and addiction-related outcomes to be examined together, and the structured tabulation of instruments, thresholds, timing, and risk of bias supports transparent comparison across studies.

At the same time, the conclusions that can be drawn are constrained by several features of the underlying literature. First, most studies relied on abbreviated screening instruments (e.g., PHQ-2, GAD-2, BSI) rather than comprehensive diagnostic assessments, limiting the ability to differentiate clearly between symptom domains and to estimate the prevalence of specific psychiatric disorders. Second, although exposure was measured in most studies, it was not consistently integrated into analytic models; this limits the ability to disentangle whether observed group differences reflect variation in exposure intensity, differential vulnerability, or both. Moreover, because exposure was characterized differently across the primary studies, reflecting each study’s own operational definitions rather than a uniform framework, direct comparison of exposure-related findings across studies is constrained. A further limitation concerns the substantial non-independence of samples. A number of the included reports recruited participants through the same commercial online survey panels (e.g., Panel4All, iPanel, Midgam), and several appear to derive from shared research programs. Because participant-level identifiers were not available, we could not determine the precise extent to which samples overlapped across reports. Recruitment through a shared panel does not necessarily indicate an identical sample, as independent samples may be drawn from the same panel at different times. Nonetheless, the possibility of partial sample overlap cannot be excluded, and the 68 reports should therefore not be assumed to represent 68 fully independent samples. The data source of each study is recorded in **Table 1** to allow readers to assess this possibility. Fourth, several populations likely to carry substantial psychiatric burden, including active-duty military personnel, military families, and children and adolescents, were comparatively underrepresented, potentially resulting in conservative estimates of overall psychiatric impact. Relatedly, the present review focused on the four symptom domains most consistently assessed in the post-October 7 literature. Other outcomes were reported too rarely to support synthesis and represent an important gap for future research. Finally, the temporal scope of the reviewed studies captures acute and early intermediate responses, providing limited insight into longer-term consequences and symptom trajectories. Sustained longitudinal follow-ups extending beyond the acute crisis period will be essential to determine whether observed symptom elevations represent transient stress reactions or consolidation into chronic psychiatric conditions requiring ongoing clinical intervention. Distinguishing between acute stress responses and persistent psychopathology has important implications for service planning, resource allocation, and the design of prevention and treatment strategies. Relatedly, all included studies derive from a single national setting and a specific phase of an ongoing conflict; accordingly, the findings may not generalize fully to other populations, conflict contexts, or health-care systems.

## Conclusion

Taken together, the reviewed evidence indicates that psychiatric responses to the October 7 attacks and the ensuing war appeared to be associated not only with exposure occurrence, but also with its continuity, accumulation, and broader social and personal context., with posttraumatic stress, anxiety, and depression frequently co-occurring and addiction-related outcomes following a more selective pattern. Distinguishing transient distress from persistent psychopathology, and symptom burden from functional impairment, will be essential for guiding service planning, resource allocation, and the design of prevention and treatment strategies in this and comparable contexts of prolonged collective crisis.

## Data Availability

The original contributions presented in the study are included in the article/**Supplementary Material**. Further inquiries can be directed to the corresponding author.

## Supplementary material

Appendix S1 Full search strategy for each database.

Appendix S2 Risk of bias assessment: item-level ratings for all included studies.
