# Supplementary material for: Psychiatric outcomes in the first two years after the October 7 attacks: a systematic review of posttraumatic stress, anxiety, depression, and addiction-related outcomes
Source: Front Psychiatry. 2026 Sep 9;17:1883498. doi: 10.3389/fpsyt.2026.1883498 (PMC13597809; doi:10.3389/fpsyt.2026.1883498)
Supplement: Supplementary file 1 [file Table1.docx]

# Appendix S1. Full Search Strategy

Searches were conducted in four databases: PubMed, PsycINFO, Scopus, and Web of Science, and limited to publications appearing from October 2023 onward, in English or Hebrew. In each database, the block of event-related terms below was combined (AND) with each of four outcome blocks, yielding four searches per database. Search terms were entered as exact phrases using Boolean operators; database-specific controlled-vocabulary mappings (e.g., MeSH in PubMed) were applied where available.

*Note. Terms such as “Israel Gaza war” and “Gaza war 2023” were included only to capture the range of labels used in the literature to denote the October 7, 2023 attacks and the ensuing war. They refer to the event, not to the study population: eligibility was restricted to Israeli populations at the screening stage, as specified in the Methods.*

**Event-related terms (combined with each outcome block below):**

("October 7" OR "October 7th" OR "October 7 2023" OR "October 7th 2023" OR "Oct 7" OR "Oct 7th" OR "October 2023 Israel" OR "Iron Swords War" OR "Swords of Iron" OR "2023 Israel Hamas" OR "Israel Hamas war 2023" OR "Israel Gaza war" OR "Gaza war 2023" OR "terror attack" OR "Hamas terror attack 2023" OR "Nova music festival attack")

**Search 1: Posttraumatic stress symptoms**

("October 7" OR "October 7th" OR "October 7 2023" OR "October 7th 2023" OR "Oct 7" OR "Oct 7th" OR "October 2023 Israel" OR "Iron Swords War" OR "Swords of Iron" OR "2023 Israel Hamas" OR "Israel Hamas war 2023" OR "Israel Gaza war" OR "Gaza war 2023" OR "terror attack" OR "Hamas terror attack 2023" OR "Nova music festival attack") AND ("PTSD" OR "PTS" OR "PTSS" OR "post traumatic symptoms" OR "post traumatic stress disorder" OR "post traumatic stress symptoms")

**Search 2: Substance use and addictive behaviors**

("October 7" OR "October 7th" OR "October 7 2023" OR "October 7th 2023" OR "Oct 7" OR "Oct 7th" OR "October 2023 Israel" OR "Iron Swords War" OR "Swords of Iron" OR "2023 Israel Hamas" OR "Israel Hamas war 2023" OR "Israel Gaza war" OR "Gaza war 2023" OR "terror attack" OR "Hamas terror attack 2023" OR "Nova music festival attack") AND ("SUD" OR "substance use disorder" OR "substance use" OR "substance abuse" OR "substance dependence" OR "addiction" OR "addictive behaviors")

**Search 3: Depression and anxiety**

("October 7" OR "October 7th" OR "October 7 2023" OR "October 7th 2023" OR "Oct 7" OR "Oct 7th" OR "October 2023 Israel" OR "Iron Swords War" OR "Swords of Iron" OR "2023 Israel Hamas" OR "Israel Hamas war 2023" OR "Israel Gaza war" OR "Gaza war 2023" OR "terror attack" OR "Hamas terror attack 2023" OR "Nova music festival attack") AND ("Depression" OR "Anxiety")

**Search 4: General mental health and psychiatric morbidity**

("October 7" OR "October 7th" OR "October 7 2023" OR "October 7th 2023" OR "Oct 7" OR "Oct 7th" OR "October 2023 Israel" OR "Iron Swords War" OR "Swords of Iron" OR "2023 Israel Hamas" OR "Israel Hamas war 2023" OR "Israel Gaza war" OR "Gaza war 2023" OR "terror attack" OR "Hamas terror attack 2023" OR "Nova music festival attack") AND ("mental health" OR "psychiatry" OR "psychopathology" OR "morbidity" OR "stress")
