# Supplementary material for: Psychiatric outcomes in the first two years after the October 7 attacks: a systematic review of posttraumatic stress, anxiety, depression, and addiction-related outcomes
Source: Front Psychiatry. 2026 Sep 9;17:1883498. doi: 10.3389/fpsyt.2026.1883498 (PMC13597809; doi:10.3389/fpsyt.2026.1883498)
Supplement: Supplementary file 2 [file Table2.docx]

| Appendix S2 | | | | | | | | | |  |
| --- | --- | --- | --- | --- | --- | --- | --- | --- | --- | --- |
| Table A1.  Critical Appraisal Checklist for Analytical Cross-Sectional Studies by the Joanna Briggs Institute (JBI) | | | | | | | | | |  |
| Study  (Author, Year) | Were the criteria for inclusion in the sample clearly defined | Were the study subjects and the setting described in detail | Was the exposure measured in a valid and reliable way | Were objective, standard criteria used for measurement of the condition? | Were confounding factors identified? | Were strategies to deal with confounding factors stated? | Were the outcomes measured in a valid and reliable way? | Was appropriate statistical analysis used? | Final | |
| Aloush et al. (2025) | Yes | Yes | Yes | Yes | Unclear | No | Unclear | Yes | Moderate |  |
| Al-Said & Braun-Lewensohn (2024) | Yes | Yes | Yes | Yes | Yes | Unclear | Yes | Unclear | Low |  |
| Ben-David et al. (2025) | Yes | Yes | Yes | Yes | Yes | Yes | Yes | Unclear | Low |  |
| Ben-Ezra et al. (2024) | Yes | Yes | Yes | Yes | Yes | Yes | Yes | Yes | Low |  |
| Bergman et al. (2025) | Yes | Yes | Yes | Yes | Yes | Unclear | Yes | Unclear | Low |  |
| Boaz et al. (2025) | Yes | Yes | Yes | Yes | Yes | Yes | Yes | Yes | Low |  |
| Dahan et al. (2024) | Yes | Yes | Yes | Yes | Yes | Yes | Yes | No | Low |  |
| Eliashar et al. (2024) | Yes | Yes | Yes | Yes | Yes | Yes | Yes | Yes | Low |  |
| Enav et al. (2025a) | Yes | Yes | Yes | Yes | Yes | Yes | Yes | Yes | Low |  |
| Enav et al. (2025b) | Yes | Yes | Yes | Yes | Yes | Yes | Yes | Yes | Low |  |
| Eshel et al. (2025) | Unclear | Yes | Unclear | Unclear | Yes | Yes | Yes | Yes | Moderate |  |
| Feingold et al. (2024) | Unclear | Yes | Yes | Yes | Yes | Yes | Unclear | Yes | Low |  |
| Haim-Nachum et al. (2025) | Yes | Yes | Yes | Yes | Yes | Yes | Yes | Yes | Low |  |
| Hamama et al. (2025) | Yes | Yes | Yes | Yes | Yes | Yes | Yes | Yes | Low |  |
| Hamama-Raz et al. (2025) | Yes | Yes | Yes | Yes | Yes | Yes | Yes | Yes | Low |  |
| Hazan-Liran & Karni-Vizer (2025) | Yes | Yes | Yes | Yes | Yes | Yes | Yes | Yes | Low |  |
| Klapper-Goldstein et al. (2025) | Yes | Yes | Yes | Yes | Yes | Yes | Yes | Yes | Low |  |
| Laufer (2024) | Yes | Yes | Yes | Unclear | Unclear | Unclear | Unclear | Unclear | High |  |
| Lipskaya-Velikovsky eal. (2025) | Yes | Yes | Yes | Yes | Yes | Yes | Yes | Yes | Low |  |
| Mayer et al. (2024) | Yes | Yes | Yes | Yes | Yes | Yes | Yes | Yes | Low |  |
| Maytles et al. (2025) | Yes | Yes | Yes | Yes | Yes | Yes | Yes | Unclear | Low |  |
| Mor et al. (2025) | Yes | Yes | Yes | Yes | Yes | Yes | Yes | Yes | Low |  |
| Palgi et al. (2024) | Yes | Yes | Yes | Yes | Yes | Yes | Yes | Unclear | Low |  |
| Peleg et al. (2025) | Yes | Yes | Yes | Yes | Unclear | Unclear | Yes | Unclear | Moderate |  |
| Pitcho et al. (2025) | Yes | Yes | Yes | Yes | Yes | Yes | Yes | Yes | Low |  |
| Rachamim et al. (2025) | Yes | Yes | Yes | Yes | Yes | Yes | Yes | Yes | Low |  |
| Reznik et al. (2025) | Yes | Yes | Unclear | Yes | Unclear | Unclear | Yes | Yes | Moderate |  |
| Ring et al. (2024) | Yes | Yes | Yes | Yes | Yes | Yes | Yes | Yes | Low |  |
| Rozenblat et al. (2024) | Yes | Yes | Yes | Yes | Yes | No | Yes | Yes | Low |  |
| Rubinstein et al. (2025) | Yes | Yes | Yes | Yes | Yes | Yes | Yes | Yes | Low |  |
| Saar-Ashkenazy et al. (2024a) | Yes | Yes | Yes | Yes | Yes | Yes | Yes | Yes | Low |  |
| Saar-Ashkenazy et al. (2024b) | Yes | Yes | Yes | Yes | Yes | Yes | Yes | Yes | Low |  |
| Saar-Ashkenazy et al. (2025) | Yes | Yes | Unclear | Yes | Unclear | Unclear | Yes | Yes | Moderate |  |
| Sarid et al. (2025) | Yes | Yes | Yes | Yes | Yes | Yes | Yes | Yes | Low |  |
| Sason et al. (2025) | Yes | Yes | Yes | Yes | Yes | Yes | Yes | Yes | Low |  |
| Sedoff et al. (2024) | Unclear | Yes | Yes | NA | Yes | Yes | Yes | Yes | Low |  |
| Shamai-Leshem et al. (2025) | Yes | Yes | Yes | Yes | Yes | Yes | Yes | Yes | Low |  |
| Shamoa-Nir (2025) | Unclear | Yes | Yes | Unclear | Yes | Yes | Yes | Yes | Low |  |
| Shapira et al. (2025). | Yes | Yes | Yes | Yes | Yes | Yes | Yes | Unclear | Low |  |
| Shechory-Bitton et al. (2024) | Yes | Unclear | Yes | Yes | Yes | Yes | Yes | Yes | Low |  |
| Shelef et al. (2025) | Yes | Yes | Yes | Yes | Yes | Unclear | Yes | Unclear | Low |  |
| Shklarski et al. (2025) | Yes | Yes | Yes | Yes | Yes | Yes | Yes | Unclear | Low |  |
| Shmulewitz et al. (2025) | Yes | Yes | Yes | Yes | Yes | Yes | Yes | Yes | Low |  |
| Shrira & Palgi (2024b) | Unclear | Yes | Yes | Unclear | Unclear | Unclear | Unclear | Unclear | High |  |
| Walter et al. (2024) | Yes | Unclear | Unclear | NA | Yes | Yes | Yes | Yes | Low |  |
| Zerach (2024) | Yes | Yes | Yes | Yes | Yes | Yes | Yes | Yes | Low |  |
| *Note*. NA = Not Applicable.  The final methodological quality rating was determined based on the number of items rated “Yes”: Low = low risk of bias (6-8 yes); Moderate = moderate risk of bias (4-5 yes); High = high risk of bias (yes ≤4). | | | | | | | | | |  |

| Table A2.  Critical Appraisal Checklist for Analytical Cohort Studies by the Joanna Briggs Institute (JBI) | | | | | | | | | | | | |
| --- | --- | --- | --- | --- | --- | --- | --- | --- | --- | --- | --- | --- |
| Study  (Author, Year) | Were the two groups similar and recruited from the same population? | Were the exposures measured similarly to assign people to both exposed and unexposed groups? | Was the exposure measured in a valid and reliable way? | Were confounding factors identified | Were strategies to deal with confounding factors stated? | Were the groups/participants free of the outcome at the start of the study (or at the moment of exposure)? | Were the outcomes measured in a valid and reliable way? | Was the follow up time reported and sufficient to be long enough for outcomes to occur? | Was follow up complete, and if not, were the reasons to loss to follow up described and explored? | Were strategies to address incomplete follow up utilized? | Was appropriate statistical analysis used? | Final |
| Aloni et al. (2025) | NA | NA | Yes | Unclear | No | Yes | Yes | Yes | Unclear | Unclear | Yes | Moderate |
| Amsalem et al. (2025a) | Yes | Yes | Yes | Yes | Yes | No | Yes | Yes | Unclear | Unclear | Yes | Low |
| Amsalem et al. (2025b) | Yes | Yes | Yes | Yes | Yes | No | Yes | Yes | No | Unclear | Yes | Low |
| Barel et al. (2025) | Yes | Yes | Yes | Yes | Yes | No | Yes | Yes | No | Yes | Yes | Low |
| Erdinast et al. (2025) | Yes | Yes | Yes | Unclear | No | No | Yes | Yes | Yes | Unclear | Yes | Moderate |
| Gilboa Schechtman et al. (2025) | Yes | Yes | Yes | Yes | Yes | No | Yes | Yes | Unclear | Unclear | Yes | Low |
| Groweiss et al. (2024) | Yes | Yes | Yes | Yes | Yes | No | Yes | Yes | Unclear | Unclear | Yes | Low |
| Levi-Belz et al. (2025a) | Yes | Yes | Yes | Yes | Yes | No | Yes | Yes | No | Unclear | Yes | Low |
| Levi-Belz et al. (2025b) | Yes | Yes | Yes | Yes | Yes | No | Yes | Yes | Yes | Yes | Yes | Low |
| Levi-Belz et al. (2025c) | Yes | Yes | Yes | Yes | Yes | No | Yes | Yes | Yes | Yes | Yes | Low |
| Levi-Belz et al. (2025d) | Yes | Yes | Yes | Yes | Yes | No | Yes | Yes | Yes | Yes | Yes | Low |
| Levi-Belz et al. (2025e) | Yes | Yes | Yes | Yes | Yes | No | Yes | Yes | Yes | Yes | Yes | Low |
| Levi-Belz et al. (2024a) | Yes | Yes | Yes | Yes | Yes | No | Yes | Yes | Yes | Yes | Yes | Low |
| Levi-Belz et al. (2024b) | Yes | Yes | Yes | Yes | Yes | No | Yes | Yes | Yes | Yes | Yes | Low |
| Levi-Belz et al. (2024c) | Yes | Yes | Yes | Yes | Yes | No | Yes | Yes | Yes | Yes | Yes | Low |
| Levi-Belz et al. (2024d) | Yes | Yes | Yes | Yes | Yes | No | Yes | Yes | Yes | Yes | Yes | Low |
| Levin et al. (2025) | Yes | Yes | Yes | Yes | Yes | No | Yes | Yes | Unclear | Unclear | Yes | Low |
| Lifshin et al. (2025) | Yes | Yes | Yes | Yes | Yes | No | Yes | Yes | No | No | Yes | Moderate |
| Palgi et al. (2025) | Yes | Yes | Yes | Yes | Yes | No | Yes | Yes | Yes | Yes | Yes | Low |
| Shrira et al. (2025) | Yes | Yes | Yes | Yes | Yes | Yes | Yes | Yes | Unclear | Yes | Yes | Low |
| Shrira et al. (2024) | Yes | Yes | Yes | Yes | Yes | Unclear | Yes | Yes | Unclear | No | Yes | Low |
| Sowan et al. (2024) | Yes | Yes | Yes | Yes | Yes | Unclear | Yes | Yes | Yes | Yes | Yes | Low |
| *Note*. NA = Not Applicable.  The final methodological quality rating was determined based on the number of items rated “Yes”: Low = low risk of bias (9-11 yes); Moderate = moderate risk of bias (6-8 yes); High = high risk of bias (yes ≤5). | | | | | | | | | | | | |
